# Construction of Konjac Glucomannan/Oxidized Hyaluronic Acid Hydrogels for Controlled Drug Release

**DOI:** 10.3390/polym14050927

**Published:** 2022-02-25

**Authors:** Hongyi Wu, Nitong Bu, Jie Chen, Yuanyuan Chen, Runzhi Sun, Chunhua Wu, Jie Pang

**Affiliations:** College of Food Science, Fujian Agriculture and Forestry University, Fuzhou 350002, China; liccfas@163.com (H.W.); bnt15850446180@163.com (N.B.); 17373679172@163.com (J.C.); chenyy6760@163.com (Y.C.); srz1206971425@163.com (R.S.)

**Keywords:** konjac glucomannan, oxidized hyaluronic acid, hydrogels

## Abstract

Konjac glucomannan (KGM) hydrogel has favorable gel-forming abilities, but its insufficient swelling capacity and poor control release characteristics limit its application. Therefore, in this study, oxidized hyaluronic acid (OHA) was used to improve the properties of KGM hydrogel. The influence of OHA on the structure and properties of KGM hydrogels was evaluated. The results show that the swelling capacity and rheological properties of the composite hydrogels increased with OHA concentration, which might be attributed to the hydrogen bond between the KGM and OHA, resulting in a compact three-dimensional gel network structure. Furthermore, epigallocatechin gallate (EGCG) was efficiently loaded into the KGM/OHA composite hydrogels and liberated in a sustained pattern. The cumulative EGCG release rate of the KGM/OHA hydrogels was enhanced by the increasing addition of OHA. The results show that the release rate of composite hydrogel can be controlled by the content of OHA. These results suggest that OHA has the potential to improve the properties and control release characteristics of KGM hydrogels.

## 1. Introduction

Hydrogels are three-dimensional polymer networks connected by cross-linked covalent bonds and weak cohesive forces in the form of hydrogen or ionic bonds [1]. Due to their excellent biocompatibility, soft texture, and high permeability to small hydrophilic molecules, hydrogels have been attractive candidates for a wide range of applications, such as cartilage scaffolds, drug delivery, and sensors [2]. Furthermore, hydrogels have been used in a variety of forms, including scaffolds, injectable hydrogels, hydrogel nanoparticles, microgels, nanofibers, and hydrogel membranes [3]. However, most hydrogels have weak stability due to their high water content, which greatly limits their application [4]. This drawback can be overcome by fabricating a “composite or hybrid hydrogel” system.

Konjac glucomannan (KGM) is a kind of natural polysaccharide that is extracted from the tuber of the Amorphophallus konjac K. Koch plant. It primarily consists of a linear chain of β-1-4-linked D-glucose and D-mannose, with a glucose to mannose ratio of approximately 1:1.6, with a low degree of acetyl groups at the side-chain C-6 position [5]. Due to its excellent film-forming ability and good biodegradable, biocompatible, and gel-forming properties [6,7], KGM has been widely applied in the field of biomedicine, food, health care, and cosmetics, among others [8,9]. In particular, KGM has been used as a raw material for the preparation of hydrogels and films that show drug-loading and sustained-release properties [5,6,9]. The preparation methods of KGM gel mainly include alkaline treatment, borate cross-linking, polymer compositing, high-voltage electric field preparation, and metal ion cross-linking after modification [6]. However, as a drug carrier, pure KGM hydrogel has a low drug release amount due to the strong hydrogen bond between KGM and other molecules [7]. Therefore, other substances need to be added to KGM hydrogel to improve its release properties.

Hyaluronic acid (HA), widely present in all organisms [10], is a nonsulfated glycosaminoglycan consisting of alternating β-1,4-D-glucuronic acid and β-1,3-N-acetyl-D-glucosamine monosaccharides [11]. Due to its non-immunogenicity and excellent degradable and excellent biocompatible properties, HA has been widely used in tissue engineering, drug delivery, and immune regulation [12]. Furthermore, as a drug carrier, it has been shown that the controlled release from HA has many benefits, such as maintaining optimum drug concentration, enhancing therapeutic effects, improving treatment efficiency with less drug, lowering toxicity, and prolonging in vivo release rates [13]. To expand the application of HA, modification is usually required. Research shows that modified HA could be mixed with chitosan and gelatin to form a hydrogel with enhanced properties [14,15]. As a modified product of HA, oxidized hyaluronic acid (OHA) has similar characteristics to HA and can be used to improve the properties of the hydrogel. The preparation of a KGM/OHA hydrogel has not been reported. In this study, HA was oxidized by sodium periodate to prepare OHA, and then mixed with KGM to form a composite hydrogel. The physical and chemical properties of the KGM/OHA composite hydrogels were evaluated by FT-IR, rheometer, and SEM. Moreover, the epigallocatechin gallate (EGCG) release properties of KGM/OHA hydrogels were also discussed. At present, the study of KGM/OHA composite hydrogels has not been reported. Therefore, this study could provide feasible advice for the preparation of KGM/OHA hydrogels and a preliminary evaluation of the properties of KGM/OHA hydrogels.

## 2. Materials and Methods

### 2.1. Materials

HA (molecular weight: 200–400 kDa), sodium periodate, EGCG, and hydroxylamine hydrochloride were purchased from Shanghai Macklin Biochemical Technology Co., Ltd. (Shanghai, China). KGM (molecular weight: 200–2000 kDa) was purchased from Zhaotong Sanai Organic Konjac Development Co., Ltd. (Yunnan, China). Glycol and anhydrous sodium carbonate were purchased from Sinopharm Chemical Reagent Co., Ltd. (Shanghai, China). The dialysis bag (M_W_ 3.5 kDa cut-off) was purchased from Shanghai Yuanye Biotechnology Co., Ltd. (Shanghai, China).

### 2.2. Preparation of OHA

OHA was prepared by the previous method with some modifications [16]. HA (1 g) was added to 100 mL of deionized water and stirred at 500 r/min for 2 h until the HA was completely dissolved. Then, 0.535 g of sodium periodate was added to the HA solution; 1mL of glycol was added to stop the reaction after 24 h of light protection. The dialysis was performed with a dialysis bag (M_W_ 3.5 kDa cut-off) for 3 d. OHA was obtained after freeze drying the dialysate for 24 h.

### 2.3. Oxidation Rate Determination

The oxidation rate was calculated according to the previous method [17]. A total of 0.05 g of OHA was added to 25 mL (0.25 mol/L) of hydroxylamine hydrochloride solution into which 2–3 drops of methyl orange were dropped. The solution was shaken for 2 h at 90 r/min. The solution of methyl orange was titrated with 0.1 mol/L of sodium hydroxide until it turned yellow. The oxidation rate can be calculated by the following formula:

(1) $$\eta =\frac{\mathrm{mol}\mathrm{of}(-CHO)}{2\cdot \mathrm{mol}\;\mathrm{of}\;\mathrm{OHA}}=\frac{V_{NaOH}\cdot N_{NaOH}\times 0.001}{2\cdot W÷400}$$

where η is the oxidation rate, $V_{NaOH}\;$ is the volume of sodium hydroxide consumed by titration (mL), $N_{NaOH}$ is the concentration of sodium hydroxide (mol/L), W is the mass of OHA (g), and 400 is the molar mass of the basic cyclic unit of OHA.

### 2.4. Preparation of Hydrogels

**Figure 1:**
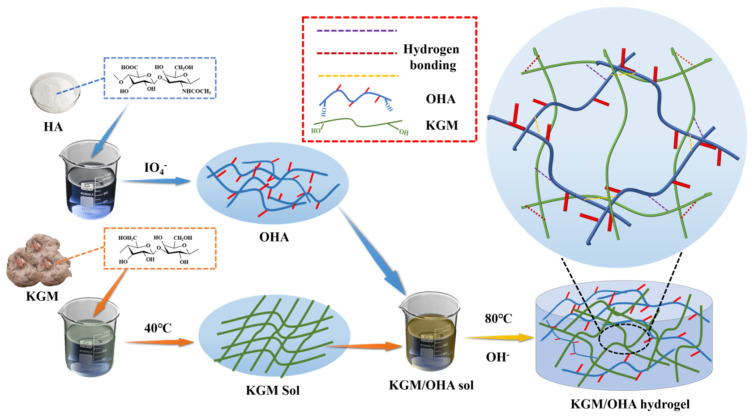
Synthesis diagram of KGM/OHA hydrogel.

The fabrication process of the hydrogels was conducted as follows: First, a certain amount of OHA was weighed and dissolved in deionized water through stirring (400 r/min) for 1 h. Then, the KGM powder was added to the OHA solution under stirring at 400 r/min. The compositions of the hydrogel samples are detailed in Table 1. After adding the KGM, 0.1 mol/L of sodium carbonate solution was added to the KGM/OHA solution to adjust the pH = 11. Subsequently, the KGM/OHA solution was placed in the water baths at 40 °C under stirring at 400 r/min for 2 h. Then, the KGM/OHA solution was transferred to the 80 °C water baths for 1 h to form hydrogel. The steps of the hydrogels’ preparation are shown in Figure 1. The obtained hydrogels were cooled to 25 °C, by running water, for further evaluation. 

### 2.5. Preparation of EGCG-Loaded Hydrogels

In order to load the EGCG into the OHA/KGM hydrogel, EGCG was directly added to deionized water at a concentration of 0.8 mg/mL, followed by a 10 min ultrasound. The freeze-dried hydrogel was then placed in the EGCG solution with 100 mL of EGCG solution for each gram of freeze-dried gel. The EGCG was loaded onto the hydrogel after standing at room temperature for 12 h [18].

### 2.6. FT-IR Characterization

The FT-IR of the OHA and KGM/OHA freeze-dried composite gels was measured by an FT-IR spectrometer (Bruker VERTEX 70, Thermo Fisher Scientific Co., Ltd., Waltham, MO, USA) using the KBr pressed pellet method. A small number of samples were mixed with potassium bromide, ground carefully for approximately 5 min, and then pressed. The FT-IR spectra were measured with wavelengths from 400 cm^−1^ to 4000 cm^−1^ with a resolution of 4 cm^−1^ and 64 scan acquisitions.

### 2.7. Rheological Test

The rheological properties of the KGM/OHA hydrogels were measured using a rotational rheometer (MCR301, Anton Parr, Austria) with a standard parallel-plate geometry (PP-50, 50 mm of diameter, and 1 mm gap). Steady shear flow behavior was measured at 25 °C and the scans were conducted at a shear rate range of 0.1 s^−1^ to 1000 s^−1^. The measurement was conducted in oscillation amplitude mode at a constant temperature of 25 °C, an angular frequency of 1.0 rad/s, and a strain range of 0.1–100%. After the linear viscoelastic region of the samples was obtained, the measurement was carried out in oscillation frequency mode, in which the strain was fixed at 1% and the frequency sweep was in a range of 0.1–100 rad/s. The values of the frequency-dependent G′ and G″ were recorded. The shear stress sweep was tested from 0.1 Pa to 100 Pa at a constant temperature of 25 °C.

### 2.8. Scanning Electron Microscopy (SEM)

The microstructures of the samples were observed through SEM (Nova NanoSEM 230, FEI CZECH REPUBLIC S.R.O., Brno, Czech Republic). The cross section of the freeze-dried gel was coated with gold and scanned at an accelerated voltage of 15 KV.

### 2.9. Swelling Properties of Hydrogels

The prepared hydrogel was freeze dried for 24 h and weighed immediately after it was taken out. The dry hydrogel was then immersed in 100 mL of PBS (0.01 M, pH = 7.4). After a certain interval of time, the hydrogel was weighed. The swelling rate was calculated as the formula:

(2) $$SD=\frac{M_{t}-M_{0}}{M_{0}}\times 100\;\%$$

where $M_{0}$ is the weight of dry hydrogel and $M_{t}$ is the weight of hydrogel after a period of time.

### 2.10. In Vitro Degradation Rate (DR)

The freeze-dried OHA/KGM hydrogels were weighed and then immersed in the PBS (0.01 M, pH = 7.4) solution at 25 °C. The hydrogels were washed with deionized water and freeze dried for 24 h. The degradation rate of hydrogel was calculated as the formula:

(3) $$DR=\frac{W_{0}-W_{t}}{W_{0}}\times 100\;\%$$

where $W_{0}$ is the initial weight of the dried hydrogel and $W_{t}$ is the weight of the dried hydrogel weighed every 4 days.

### 2.11. EGCG Loading Determination

The concentration of EGCG was measured using the Folin-Ciocalteu phenol method [19]. The absorbance of the solution was recorded at a 717 nm wavelength using a UV–vis spectrophotometer (UV-1780, Shimadzu, Japan). The concentration and content of EGCG in the solution were calculated by the standard curve of EGCG absorbance. The entrapment efficiencies (EEs) of the KGM/OHA hydrogel for EGCG were determined using the following equation:

(4) $$\mathrm{EEs}=\frac{m_{0}-m_{1}}{m_{0}}\times 100\%$$

where *m*_0_ is the total amount of EGCG contained in the suspension and *m*_1_ is the amount of free EGCG.

### 2.12. In Vitro Release Studies

In order to evaluate the sustained release performance of KGM/OHA hydrogel, the KGM/OHA hydrogel, loaded with EGCG, was immersed in 50 mL of PBS (0.01 M, pH = 7.4) and continuously shaken at 60× *g* rpm. The temperature was maintained at 37 °C. Then, 1 mL of the solution was taken out every 1 h to measure the content of EGCG, and the release amount of EGCG was, also, calculated according to the Folin–Ciocalteu phenol method. The concentration and content of EGCG in the PBS were calculated by the standard curve of EGCG absorbance. The cumulative release rate of EGCG was calculated by the EGCG content in the hydrogel and the solution before the release experiment. The formula is as follows:

(5) $$Cumulative\;release\;rate=\frac{m_{t}}{m_{0}-m_{1}}\times 100\%$$

where *m_t_* is the EGCG content in the PBS solution after a certain release time. The volume of PBS was kept at 50 mL. The experimental data were obtained from three parallel experiments.

## 3. Result and Discussion

### 3.1. Characterization of OHA

**Figure 2:**
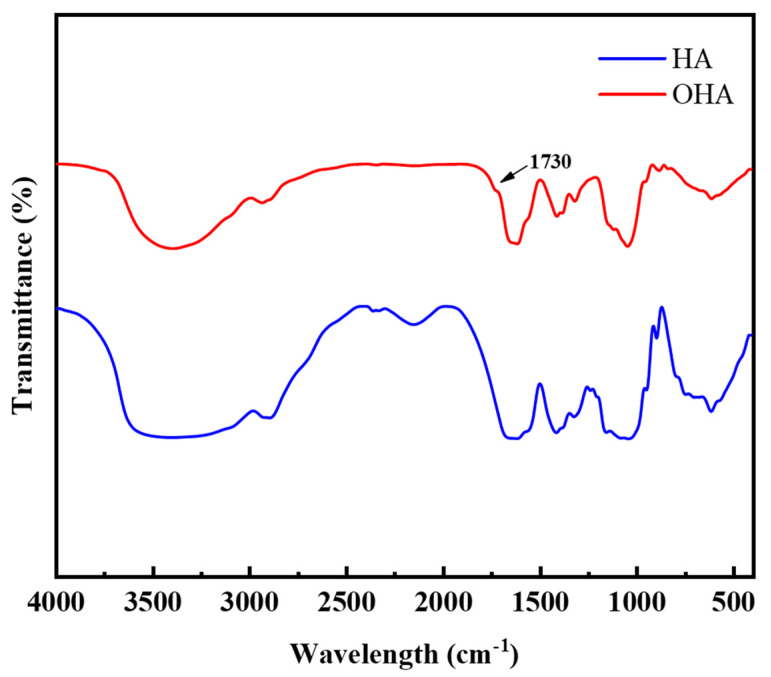
FT-IR spectra of HA and OHA.

Sodium periodate can oxidize two adjacent hydroxyl groups in HA into two aldehyde groups to form OHA. Because the amino group in hydroxylamine hydrochloride can react with the aldehyde group in OHA, the reaction between hydroxylamine hydrochloride and the aldehyde group can be used to verify whether OHA can be successfully prepared [20]. The content of the aldehyde group in OHA was calculated according to the reaction formula, and the oxidation rate of OHA was 43.8 ± 2.26%, which was consistent with previous research [21]. To further confirm the successful formation of the dialdehyde groups in OHA, FT-IR spectra of HA and OHA were recorded. As shown in Figure 2, compared with HA, a new absorption peak at approximately 1725 cm^−1^, corresponding to the dialdehyde groups, was observed in the spectrum of OHA [15], which confirmed the successful preparation of OHA. Similar results were also reported by Li et al. [22].

### 3.2. Appearance Characteristics of Hydrogels

**Figure 3:**
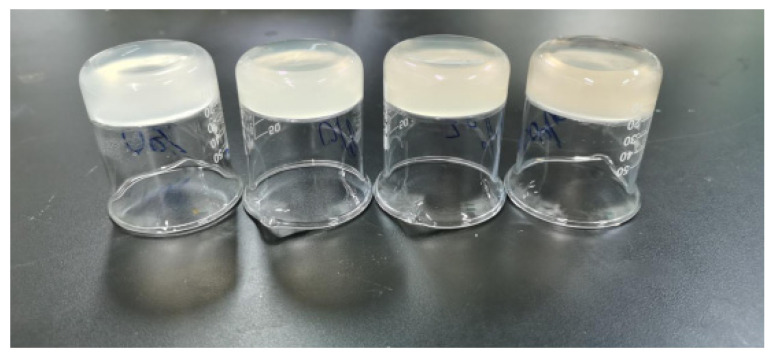
An image of the hydrogels. From left to right, the hydrogels are KO-0, KO-1, KO-2 and KO-3, respectively.

The photographic appearance of the hydrogels is shown in Figure 3. It was obvious that all of the hydrogels were pale yellow due to the deacetylation of KGM during the formation of the hydrogels [23]. The original slight yellow color of the OHA hydrogel might be responsible for this. With the increase in the OHA concentration, the composite hydrogels’ color became deeper. Meanwhile, the structure of all the hydrogels was flat without collapse, indicating the good potential of KGM and OHA for the formulation of hydrogels.

### 3.3. FT-IR Spectra of Hydrogels

**Figure 4:**
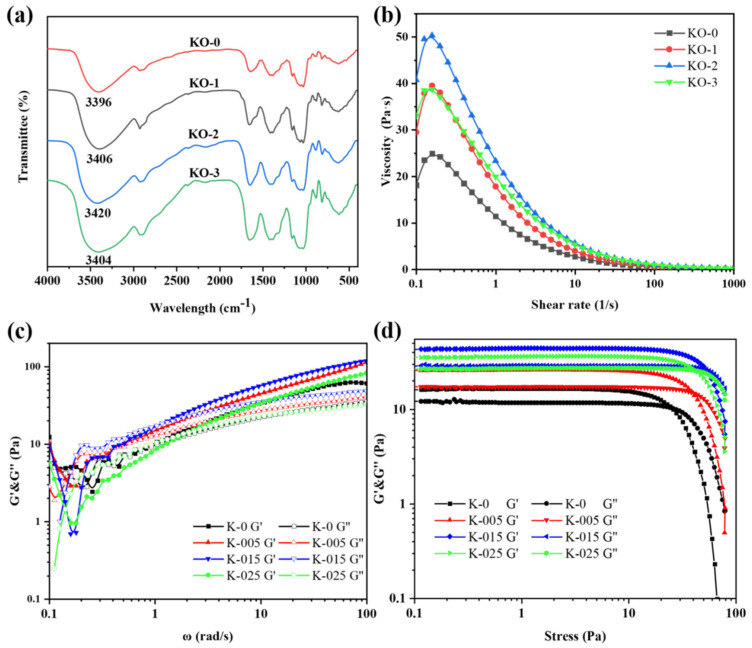
FT-IR spectra of KO-0 hydrogel, KO-1 hydrogel, KO-2 hydrogel and KO-3 hydrogel (**a**). The viscosity–shear rate curves (**b**), the viscoelasticity–angular frequency curves (**c**) and the viscoelasticity–shear stress curves (**d**) of KO-0, KO-1, KO-2 and KO-3 hydrogels.

FT-IR was usually used to observe functional groups in polysaccharides. Changes occurring at a molecular level were often difficult to observe from a macroscopic perspective. The FT-IR spectra of the KO-0, KO-1, KO-2 and KO-3 hydrogels are shown in Figure 4a. The spectra of the KO-0, KO-1, KO-2 and KO-3 hydrogels were basically similar. However, the O-H bond absorption peak near 3406 cm^−1^ moved with the addition of OHA. The peaks of KO-0, KO-1, KO-2 and KO-3 appeared at 3396 cm^−1^, 3406 cm^−1^, 3420 cm^−1^, and 3404 cm^−1^, respectively. From KO-0 to KO-2, the site of the O-H bond absorption peak gradually deviated to the high wavelength due to the stretching of the O-H bond. This was attributed to the hydrogen bond interaction between the KGM/OHA hydrogels that gradually increased with the increase in the OHA content [24]. However, the hydrogen bond absorption peak site of the KO-3 hydrogel was close to that of KO-1, indicating that the hydrogen bond interaction of KGM/OHA hydrogel was weakened when the OHA concentration was 0.5%. 

### 3.4. Rheological Analysis of Hydrogels

To investigate the effect of the OHA amount on the rheological properties of composite hydrogels, the steady rheological behavior of the composite hydrogels with different OHA contents was measured, as shown in Figure 4b. It was found that the apparent viscosity of all the hydrogels could be maintained at a relatively high level at a low shear rate. With the increase in shear rate, the apparent viscosity of the hydrogels gradually decreased and finally stabilized at an almost identical low viscosity level. It shows that the properties of pseudoplastic fluid were consistent with the rheological properties of KGM studied previously [25]. At a lower shear rate, the three-dimensional network structure of the hydrogels was not damaged, so the viscosity of the hydrogels could be maintained at a high level. However, with the increase in shear rate, the molecular chain gradually fractured, leading to an increased degree of damage to the three-dimensional network of the hydrogels and a decrease in the viscosity of the hydrogels [26]. In addition, adding OHA can significantly improve the viscosity of the mixed system, which might be due to the hydrogen-bond interaction between the OHA and KGM that made the hydrogels’ network structure more stable. Among the three hydrogels containing OHA, the hydrogels with an OHA concentration of 0.3% had the highest viscosity. This was because the main effect of the OHA addition on the KGM hydrogel was to reinforce the network structure at a concentration of 0–0.3%. However, the viscosity of the hydrogel decreased at an OHA concentration of 0.5%. This may be because the excessive OHA addition might have impacted the original structure of the KGM gel, allowing the molecules to slide.

Frequency scanning is one of the most common test modes in polymer dynamic rheological testing. Through frequency scanning, the frequency dependence of the storage modulus (G′) and loss modulus (G″) can be obtained in order, so as to judge the relaxation time of the polymers. The relationship between the viscoelasticity and angular frequency of the hydrogels is shown in Figure 4c. G′ represents the elasticity of hydrogel while G″ represents the viscosity of the hydrogel. It has been previously stated that when the G′ value of a substance is greater than the G″ value, the substance acts as a viscoelastic solid, indicating that the substance behaves like a gel [25,27]. It can be seen that the G′ values of all the hydrogels were higher than the G″ values in the whole angular frequency measurement range (0.1–100 rad/s), and all the hydrogels exhibited stable viscoelastic solid morphology. These results indicate that hydrogels can keep their original hydrogel network structure in a middle frequency band and have good structural stability. In addition, the viscoelasticity of all the hydrogels containing OHA was higher than that of the KGM hydrogels, indicating that OHA could improve the stability of the KGM hydrogel network. This result was consistent with the steady rheological study. The hydrogels with an OHA concentration of 0.3% still had the highest viscoelasticity, which was consistent with the performance of viscosity and the shear rate of the hydrogels.

The mechanical properties of the hydrogels reflected the homogeneity state and the interfacial interactions between its components, which was measured by oscillatory shear rheology [27]. The hydrogels behaved as viscoelastic fluids under a certain degree of shear stress, and the critical value of shear stress was also shown as the intersection of the G′ and G″ curves [28]. As shown in Figure 4d, the viscoelasticity of the KGM hydrogel was lower than that of the hydrogel containing OHA. The intersection point of the G′ and G″ curves of the KGM hydrogel was more advanced than those of the other hydrogels containing OHA. These results demonstrate that the mechanical properties of the KGM hydrogels were significantly lower than those of the hydrogels containing OHA [26], indicating that OHA could improve the mechanical properties of hydrogel and make the hydrogel structure more stable. Furthermore, the hydrogel structure at an OHA concentration of 0.5% was weaker, which was consistent with the above experiments.

### 3.5. SEM Analysis

**Figure 5:**
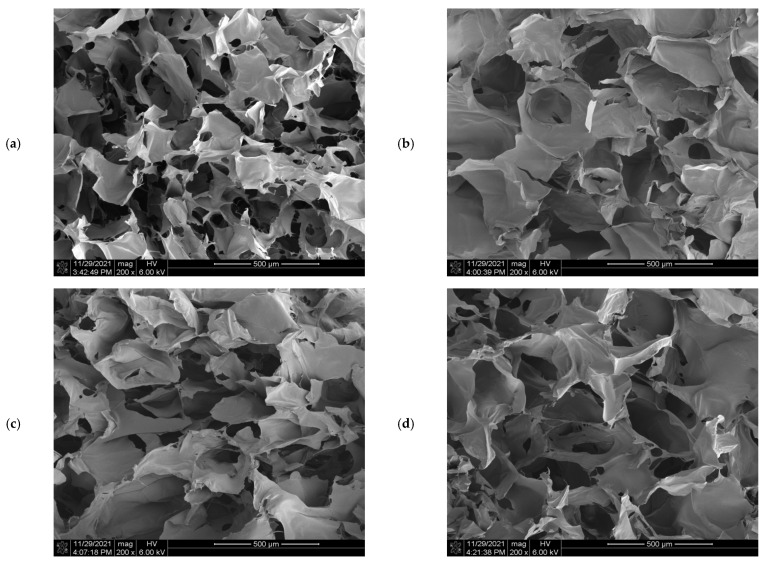
The SEM images of KO-0 hydrogels (**a**), KO-1 hydrogels (**b**), KO-2 hydrogels (**c**) and KO-3 hydrogels (**d**).

Figure 5 shows the morphology of the KGM and KGM/OHA freeze-dried hydrogels with different OHA concentrations. It can be seen that all the hydrogels present continuous network structures, which was the structural characteristic of stable hydrogels. This was similar to the observation of the KGM hydrogel network structure under alkaline conditions by Mu et al. [29]. It indicated that the network structure of KGM occupied the majority in the gel system of KGM/OHA, which corresponded to the higher content of KGM relative to OHA in the experimental group design. In addition, the network structure of the KGM/OHA hydrogels was more compact than that of the KGM hydrogels after adding OHA. It can be speculated that the OHA molecules wound onto the KGM gel network, and its molecular chains were connected by the hydrogen bond interaction, which strengthened the network structure of the KGM hydrogel and thus enhanced the stability of the KGM hydrogel. However, when the content of OHA was 0.5%, due to the hydrophilicity of OHA, a large number of holes appeared in the network structure of the KGM/OHA hydrogel after freeze drying [30]. Adding too much OHA increased the water content within the hydrogel, which led to many water-filled holes in the structure of the hydrogel. Therefore, the excessive OHA made the water content of the hydrogel higher, thereby resulting in a decrease in the viscosity and stability of the KGM/OHA hydrogel at 0.5% OHA content.

### 3.6. Swelling Properties

**Figure 6:**
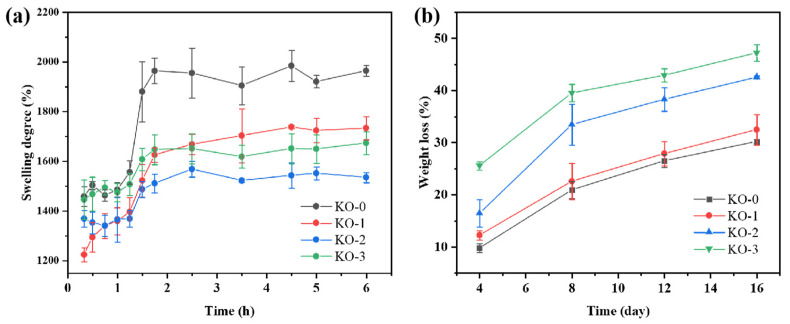
Swelling curve of KO-0 hydrogels, KO-1 hydrogels, KO-2 hydrogels, and KO-3 hydrogels (**a**). In vitro degradation curve of KO-0 hydrogels, KO-1 hydrogels, KO-2 hydrogels, and KO-3 hydrogels (**b**).

Swelling is one of the main properties of hydrogels. The swelling properties of the hydrogel within 6h are shown in Figure 6a. It can be observed that the swelling rate of all the prepared hydrogels increased, with the increase in soaking time, and finally reached a relatively stable level. It indicated that the hydrogels could swell well in the PBS solution with a pH of 7.4, which was similar to the swelling trend of the KGM hydrogels observed previously [31]. In addition, it can be seen, from the broken line diagram of gel swelling, that the swelling rate of the KGM hydrogel was higher than that of the KGM/OHA hydrogel. The swelling rate of the hydrogels was usually related to the cross-linking density and the network structure of the hydrogels [32]. As the cross-linking density increased, the swelling rate of the hydrogels decreased, which was probably due to the denser network formed in the hydrogels with a higher cross-linking density, thus making it difficult for water molecules to enter the internal space of the hydrogels [33]. Therefore, with the addition of OHA, the swelling rate of the hydrogels became lower because the cross-linking density of the KGM hydrogel was lower than that of the KGM/OHA hydrogel. When the OHA concentration ranged from 0 to 0.3%, the decreasing swelling rate showed that the cross-linking density of the hydrogel network gradually increased. However, when the OHA concentration was 0.5%, excessive OHA made the microstructure of the hydrogel appear porous, which reduced the cross-linking density of the hydrogels and increased the swelling rate.

### 3.7. In Vitro Degradation Rate

In order to study the degradation performance of hydrogels, the hydrogels were soaked in PBS solution and then removed every 4 days for drying and weighing, followed by a recording of the mass loss of the hydrogels, which can be utilized for reflecting the degradation rate of the hydrogels. The degradation rate of the hydrogels in different proportions is shown in Figure 6b. The results show that the degradation rate of the hydrogel increased gradually within 16 d, which was a common phenomenon of hydrogel degradation in vitro. In addition, the degradation rate of hydrogels containing OHA was significantly higher than that of the KGM hydrogels within 16 d, and the degradation rate of hydrogels in vitro increased with the increase in the OHA supplemental level. This phenomenon was due to the high degradation capacity of the OHA [33]. The cyclic monomer of the OHA contained an aldehyde group, which was beneficial to degradation [34]. Therefore, the addition of OHA could make KGM hydrogel possess a higher degradation rate.

### 3.8. Load and Release Behavior of Hydrogels

The entrapment efficiencies (EEs) of KO-0, KO-1, KO-2, and KO-3 hydrogels were 18.43 ± 1.99%, 19.13 ± 1.94%, 22.40 ± 1.24%, and 20.87 ± 2.28%, respectively. The EEs of the hydrogels were usually related to the microstructure of the hydrogel network. When the network structure of the hydrogels was relatively loose, the area available for EGCG attachment was less [35]. The increase in the number of pores in the hydrogel could also improve EEs, which was based on the principle that the pores could increase the internal surface area of the hydrogel [36]. SEM showed that from KO-0 to KO-2, the network of hydrogels became more compact. KO-2 hydrogels had the densest network structure and contained many pores and, thus, had the highest EEs. Compared with the KO-2 hydrogel, the KO-3 hydrogel had slightly more pores, but the excessive OHA impacted the molecular structure of the KGM and made the structure of the hydrogel not compact. Moreover, the reduced load space of EGCG on the hydrogel resulted in the EEs of the KO-3 hydrogel being slightly lower than that of the KO-2 hydrogel. 

**Figure 7:**
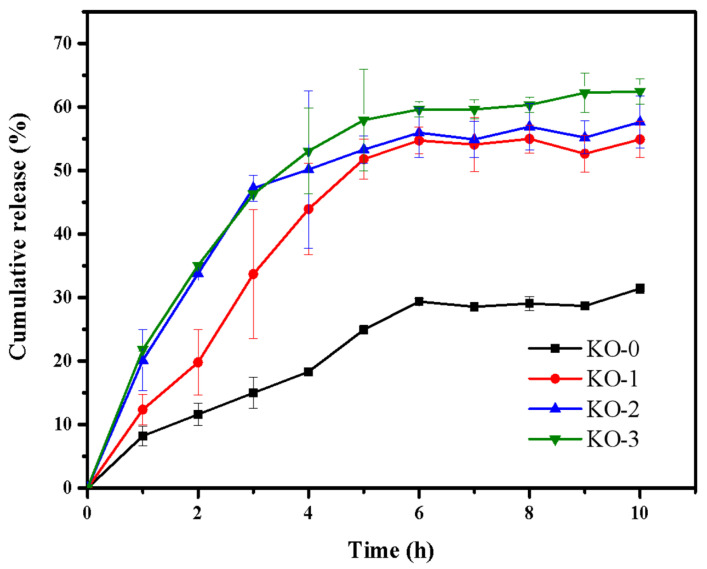
Cumulative release curves of KO-0, KO-1, KO-2 and KO-3 hydrogels.

Figure 7 shows the EGCG release rates of KGM/OHA hydrogels in different proportions. The EGCG release amounts of KO-0, KO-1, KO-2, and KO-3 hydrogels after 10 h were 31.40 ± 0.73%, 54.89 ± 2.81%, 57.62 ± 4.11%, and 62.44 ± 1.97%, respectively. Figure 7 also shows that the amount of EGCG released in the first 6 h was significantly higher than that in the following 4 h. The phenomenon observed by Wang et al. [37] and Yuan et al. [38] did not appear in this experiment: hydrogels showed an explosive release of the loaded substance in the first 1 h of the release experiment and a sustained release in the following several hours. In this experiment, all hydrogels showed higher release rates in the first 5 h of the release experiment than in the subsequent hours. Although the burst release rate was lower, KGM/OHA hydrogels showed a longer burst release period of EGCG than other KGM-based hydrogels. This phenomenon was attributed to the fact that OHA could prolong the in vivo release [13]. The other reason was that the molecular structure of EGCG contained a large number of hydroxyl groups so that it was easy to have a strong hydrogen bond interaction with the hydroxyl groups on the KGM and OHA molecular chains [39]. The gradual increase in the EGCG release of the KO-1, KO-2, and KO-3 hydrogels was due to the hydrophilicity and degradation of the OHA [30,34]. The hydrophilic properties of the OHA allowed water to enter the internal structure of the KGM/OHA hydrogels more quickly, which allowed the EGCG in the hydrogel to be released into the buffer more easily. The rapid degradation of the OHA made the structure of the gel become gradually fluffy during the release experiment, which made EGCG easy to release. It could be concluded that the release rate and amount were related to the content of OHA and that they can be easily controlled by the addition of OHA. Therefore, KGM/OHA hydrogels, capable of the long-term, high-speed, and controlled release of EGCG, can be used for biomedical and food applications, as a promising gel material.

## 4. Conclusions

In this study, a stable composite hydrogel was prepared by incorporating KGM with OHA, after which alkali processing and thermal treatment were conducted. The effect of OHA content on various gel properties was evaluated. The obtained hydrogel was pale yellow, smooth in surface, and had a favorable swelling capacity, which qualified the essential requirements for ideal drug-delivery applications. The OHA played an effective role in adjusting the swelling ratio and increasing the biodegradation rate. The rheological analysis shows that, when the concentration of OHA is 0.3%, KGM/OHA has the most stable network structure and the greatest mechanical properties. When observed through SEM, the firm and porous structure of the KGM/OHA hydrogel demonstrates a rich storage space for drug loading. Furthermore, both EGCG encapsulation efficiency and the release properties of the hydrogels were significantly raised with the presence of OHA. The overall results suggest that the KGM/OHA hydrogel, loaded with EGCG, exhibited potential applications in controlled release.

## Figures and Tables

**Table 1:** Composition of KGM and OHA in hydrogels.

Sample	KO-0	KO-1	KO-2	KO-3
KGM% (*w*/*v*)	1	1	1	1
OHA% (*w*/*v*)	0	0.1	0.3	0.5

## Data Availability

Data are contained within the article.
